# Sensors Special Issue: “Vibration Energy Harvesting for Wireless Sensors”

**DOI:** 10.3390/s22124578

**Published:** 2022-06-17

**Authors:** Zdenek Hadas, Saša Zelenika, Vikram Pakrashi

**Affiliations:** 1 Faculty of Mechanical Engineering, Brno University of Technology, 616 69 Brno, Czech Republic; 2 University of Rijeka, Faculty of Engineering & Centre for Micro- and Nanosciences and Technologies, Vukovarska 58, 51000 Rijeka, Croatia; 3 UCD Centre for Mechanics, Dynamical Systems and Risk Laboratory, School of Mechanical and Materials Engineering, University College Dublin, D04 V1W8 Dublin, Ireland

Mechanical vibrations occur in the operation of most technical systems. High-level vibrations could indicate an overloaded or damaged technical system. These states and behaviours can be monitored or reported, and ambient vibrations may, in turn, be used as a source of energy. The vibrational energy harvesters used in this framework could be an alternative to the supply of low-power autonomous electronic systems for the remote sensing of operations. However, the level of energy harvested using such an approach is usually very low, and the whole concept of vibration energy harvesting system operations (including power management electronics and wireless sensors) must be adapted for specific target applications.

This Special Issue, through twelve diverse contributions, intends to present some of the contemporary challenges, solutions and insights around the outlined issues and provides an overview of this rapidly evolving topic. The papers collected in this SI cover a variety of energy harvesting sources, as well as the need to create bases of numerical and experimental evidence around them. Qualifying and quantifying their performances in terms of energy harvesting levels, as well as their consistency and potential applications, are all reflected in these papers. The fundamental importance of understanding sensors, along with their possible integration within their respective application areas, including those related to the effective communication of measurement data, are also emphasized throughout this Special Issue.

Mech et al. [1] studied magnetomechanical harvesting and the related possibility of data transfer, investigating a novel hybrid solution focusing on rapid demagnetization [2]. Hadas et al. [3] explored an electromagnetic harvester for railway applications, where energy is transferred from the vibration of the rails during train operations, thereby leading to applications that can encompass monitoring. Litak et al. [4], on the other hand, focused on the fundamentals of energy harvesting and investigated aspects of bifurcation caused by their nonlinearities, whereby different domains of harvesting exist. In particular, the impact of hysteresis was analysed, a topic that should be explored in more detail by the energy harvesting scientific community. Koszewnik et al. [5] developed a smart beam made from macro fiber composites (MFC), numerically and experimentally demonstrating how energy harvesting can be used for damage detection in structural health monitoring. Machu et al. [6] further investigated the design of energy-harvesting-powered sensors through various configurations, creating experimental verifications of analytical models, which makes it possible to develop robust models with minimized computational effort, in-keeping with fundamental physics. Kunz et al. [7] focused their efforts on novel methods to assess the performance of these harvesters in terms of power flow. Bae and Kim [8], on the other hand, approached sensor performance issues in terms of load resistance optimisation for a bi-stable system. Okosun et al. [9] addressed one of the core sustainable development goals, the availability of drinking water, and experimentally demonstrated how energy harvesting patches can be used for pipeline leak detection, creating a respective benchmark. The topic of experimental validation was developed by Chen et al. [10], who studied the influence of driven harvesters in a magnetic field. For these harvesters, their source is important, and a comparison between piezoelectric and triboelectric harvesting of energy is investigated by Thainiramit et al. [11]. Finally, Phan et al. [12] provided a short and impactful investigation of electromagnetic harvesters with linear and nonlinear springs.

The dynamism and breadth of the treated topic is clearly demonstrated by the contributions to this Special Issue, as is the variety of approaches that are available. We expect this field of study to move further towards an interdisciplinary research field in the near future, giving rise to new sensors, methods of measurement and impactful applications across a range of sectors, established through scientific rigour, along with numerical and experimental benchmarks.

## Data Availability

Not applicable.
